# Contagious Effect of Nurses' Perception of Leaders' Antisocial Behaviour

**DOI:** 10.1111/jan.70381

**Published:** 2025-11-11

**Authors:** Kwadwo Asante, Petr Novak, Konadu Angela Achiaa, Torben Juul Andersen

**Affiliations:** ^1^ Tomas Bata University in Zlin Zlin Czech Republic; ^2^ Queen Elizabeth University Hospital Glasgow UK; ^3^ Copenhagen Business School Department of International Economics and Management Frederiksberg Denmark

**Keywords:** antisocial behaviour, Ghana, healthcare workers, need for recognition, nurses, personal norm, smart‐PLS, social contagion

## Abstract

**Aim:**

To examine the underlying mechanism that strengthens or attenuates the social contagion effect among nursing professionals.

**Design:**

The study uses a cross‐sectional design. The study's results followed the Strengthening Reporting of Observational Studies in Epidemiology (STROBE).

**Methods:**

A Questionnaire was used as the main source of data collection. The data collection occurred between March 11 and May 12, 2024. The study used purposive sampling to select 25 health facilities. A total of 530 questionnaires were sent out, of which 323 responses were received, and 27 were excluded due to missing data and logical inconsistency. In all, 296 responses were used for the analysis, giving a valid response rate of 58.8%. The smart partial least squares partial equation modelling (Smart‐PLS 4.0) was used for the study's data analysis.

**Results:**

The results reveal that the need for recognition mediates the relationship between nursing managers' and subordinates' antisocial behaviour. Also, results from the study indicate that personal norm inversely moderated the relationship between superior antisocial behaviour and subordinate behaviour.

**Conclusion:**

The impact of superior antisocial behaviour on junior nurses may not translate into the same level of effect, especially when the nurse perceives her role as a call to duty (i.e., has high personal norms).

**Impact:**

The study findings confirm the crucial role personal norms and the need for recognition play in strengthening or weakening the social contagion effect of senior nurses' antisocial behaviour on junior nurses' behaviour.

**Reporting Method:**

The study followed the Strengthening Reporting of Observational Studies in Epidemiology (STROBE) guidelines.

**Patient or Public Contribution:**

No patient or public contribution.

## 1 Introduction

Nursing managers are strategically positioned in the healthcare system to create positive experiences, such as serving as positive role models and inspiring nurses to advance in their roles (Havens 2011). It has long been recognised that nursing managers who provide the needed leadership experience can shape junior nurses' work behaviours in diverse ways, from job satisfaction, commitment, turnover decisions and citizenship behaviour (Belbin et al. 2012; Feather 2015). Therefore, nursing managers' leadership principles based on respect, trust, and effective communication have become imperative for creating a working environment where nurses become well‐motivated and adequately engaged in their work roles (Field and Brown 2019).

Notwithstanding nursing managers' expected influence on junior nurses' behaviour, the outcomes of social interactions between senior and junior nurses within the healthcare setting do not always lead to a positive outcome (Zheng et al. 2017). For instance, nurses who work under abusive superiors tend to perform poorly in their work roles (Low et al. 2021) and demonstrate high job disengagement (Yang et al. 2017) and weak organisational citizenship behaviour (Lyu et al. 2019). This phenomenon, called social contagion (Levy and Nail 1993), constitutes the unintended spillover of a person's behaviours, attitudes, and ideals to people within the same proximity in a social setting. Social contagion has been seen to occur in various aspects of our lives, from motor skills to emotions, attitudes and risk‐taking (Reiter et al. 2019; Burgess et al. 2020). Because a person has a high propensity to align with the views of a superior, especially when the leader is perceived as powerful, the effect of social contagion on individual behaviour and attitude becomes inevitable (Zaki et al. 2011; Lindenberg et al. 2021).

Moreover, considering that social contagion spillover tends to be much higher in a work setting characterised by a high frequency of engagements, there have been calls for scholars to explore its effect among nursing professionals (Ogunlade 1979; Dimant 2019; Shafique et al. 2020). Unlike other professions, there is high social proximity between senior and junior nurses, resulting in the unintended consequences of the leader's influence on subordinate behaviour (Dimant 2019). Admittedly, the growing body of studies has confirmed the reoccurrence of the contagion influence of leaders' behaviour on subordinates such as withdrawal behaviour (Eder and Eisenberger 2008), safety violations (Liang et al. 2018), employee turnover (Porter and Rigby 2021) and knowledge sabotage in the workplace (Serenko and Abubakar 2023). Nonetheless, only a few studies have been conducted among nursing professionals (Shafique et al. 2020), with many of the studies emphasising its effect as permanently unidimensional (Inam et al. 2021; Liu et al. 2021).

However, because quality healthcare service is better achieved through health workers' pro‐social behaviour (Asante 2025), nurses' antisocial behaviour, which is described as the negative behaviours including abuse, withdrawal from responsibilities, sabotage, non‐supportive and self‐centred behaviours (Liang et al. 2018), becomes a threat to quality healthcare delivery (Zhuang et al. 2020). It therefore becomes imperative for healthcare institutions to minimise the occurrence of antisocial behaviour at the health facility (Lian et al. 2014) and determine the factors that influence its occurrence (Lugosi 2019).

## 2 Background

Ghana's health sector promotes easy access to primary healthcare. It is one of the few countries in Sub‐Saharan Africa with over 100,000 healthcare workers (World Health Organisation 2021). Even though Ghana is a strong supporter of the Sustainable Development Goals (SDGs), like many other low‐ and middle‐income countries, its health sector is still not in strict compliance with Goal 3—good health and well‐being, Goal 8—decent work and economic growth, and Goal 11—reduced inequalities of the SDGs (United Nations 2023). For instance, unlike the developed economies, where the nurse‐to‐patient ratio aligns with the World Health Organisation Standard, Ghana's nurse‐to‐patient ratio stands at 1:1839 (World Health Organisation 2021). Aside from this shortfall in the country's nursing population, there has also been a surge in the relocation of senior nurses to economies such as the United Kingdom and the United States in search of better conditions of service (Ibrahim et al. 2024). Again, most Ghanaian nurses work in harsh conditions, affecting their ability to deliver their services effectively, particularly in rural communities (Adu‐Gyamfi and Brenya 2016). Additionally, as most Ghanaian hospitals are under‐resourced and overburdened with more cases, nurses are likely to engage in antisocial behaviours to safeguard themselves from these workplace stressors (Vukey et al. 2023). Accordingly, understanding how social contagion diffuses from senior nurses to junior nurses in under‐resourced facilities will help identify the conditions that strengthen this condition and give a different perspective on what individual‐level factors can counteract this negative social influence in a strong, socially dependent setting.

Although the studies on social contagion are growing (Hsieh et al. 2020; Inam et al. 2021; Liu et al. 2021), only a few of these studies have been conducted in the nursing context, thereby limiting our understanding of the specific conditions that exacerbate or attenuate this relationship among nursing professionals (Shafique et al. 2020; Koroglu and Öksüz 2024). For instance, Shafique et al. (2020) found that nurses who witnessed more ostracism from their nursing managers reported high engagement in deviant behaviour. On the other hand, Koroglu and Öksüz (2024) explored the level of emotional contagion and spiritual care competencies among intensive care nurses. Results from their study suggest that intensive care nurses are highly vulnerable to emotional contagion within their work unit.

Despite the few studies among nursing, most of these extant works have assumed that the relationship between social contagion and employee outcomes is always unidimensional (Mayer et al. 2009; Cialdini et al. 2021). However, some evidence suggests that a person's values and ideals could intensify or weaken the social contagion impact, indicating that the expected effect cannot always be homogeneous (Zhang et al. 2019; Jeworrek and Waibel 2021). Therefore, there are gaps in the literature that need to be addressed. First, considering that a nurse may care more about the social suitability of their actions (Krupka and Weber 2013), individual personalities such as norms and values become essential attributes that can thwart the contagious effect of leadership influence on junior nurses' antisocial behaviour (Kish‐Gephart et al. 2010). For instance, personal values enable people to define what is good, worthy and critical in their life (Kmieciak 2024). It becomes the far‐reaching, trans‐situational, wanted goals that act as a signpost in a person's actions and help them define right and wrong (Sagiv et al. 2017, 630). According to Iqbal (2021), considering the level of importance people attach to their values, it tends to substantially impact individual life choices, attitudes and workplace behaviour (Dalvi‐Esfahani et al. 2017). Therefore, drawing on the person‐organisation misfit (P‐O) (Cable and Edwards 2004) and norm activation model (NAM) (Schwartz 1977) arguments, we argue that a person can be differentially well matched in a given organisational setting based on the non‐alignment between their personal values and organisational actions. However, how personal values affect the social contagion effect among superiors and employees remains empirically untested.

Secondly, though we argued that solid personal norms can weaken the social contagion of superior antisocial behaviour (Schwartz 1977), the high subordinate dependence on superiors for their survival and career growth, particularly in developing economies, raises doubts about how a nurse's values may affect leadership social contagion. To attract a positive favour from their superiors, subordinates will imitate their managers' behaviours at the workplace (Mawritz et al. 2012). The excessive desire to gain favours from superiors may stimulate employees to neglect their personal moral principles in pursuit of leadership ideals (Umphress and Bingham 2011). Therefore, we argued that the need for recognition (NFR), which is described as the desire to gain recognition or praise for an accomplishment (Keluskar 2023), may weaken individual values by giving nurses a heightened incentive to follow through with the antisocial behaviours of their superiors to acquire favours and benefits (Banerjee and Duflo 2012). Also, since nurses operate in an environment characterised by high reverence for superiors (Asante 2025), high personal values may not be sufficient to inoculate a nurse against the social contagion of their superior influence. Empirically, it is unclear what type of social contagion will occur: will a strong personal norm of the nurse suppress the contagious effect of the supervisors' antisocial behaviour, or will the strong desire for recognition from superiors exacerbate social contagion? The study addresses these gaps in the literature by posing the following research hypotheses:

Hypothesis 1 *Superior antisocial behaviour influences subordinates' antisocial behaviour*.

Hypothesis 2 *Superior antisocial* behaviour *directly affects subordinates' need for recognition*.

Hypothesis 3 *Need for recognition mediates the relationship between superior and subordinate antisocial* behaviour.

Hypothesis 4 *Need for recognition directly influences subordinates' antisocial behaviour*.

Hypothesis 5 *Personal norm inversely moderates the relationship between superior and subordinate antisocial behaviour*.

Hypothesis 6 *Personal norm inversely moderates the relationship between superior antisocial behaviour and need for recognition*.

**FIGURE 1:**
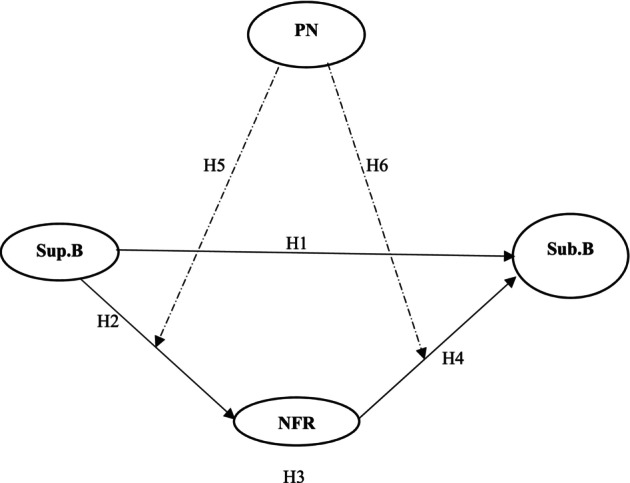
Proposed conceptual framework. ‐‐‐‐‐ Moderation; —— Direct/mediation. H1: Superior antisocial behaviour influences subordinates' antisocial behaviour. H2: Superior antisocial behaviour directly affects subordinates' need for recognition. H3: Need for recognition mediates the relationship between superior and subordinate antisocial behaviour. H4: Need for recognition directly influences subordinates' antisocial behaviour. H5: Personal norm inversely moderates the relationship between superior and subordinate antisocial behaviour. H6. Personal norm inversely moderates the relationship between superior antisocial behaviour and need for recognition. NFR, need for recognition; PN, personal norm; Sub B, subordinate antisocial behaviour; Sup B, superior antisocial behaviour.

The study draws on the lens of the SIP (Zalesny and Ford 1990) and the NAM (Schwartz 1977) to introduce two social constructs, personal norms and the NFR, to understand the underlying mechanisms that affect social contagion on nurses' behaviour in a developing economy. Investigating the role of personal norms and the NFR in social contagion relationships will provide a new understanding of the antecedents that strengthen or weaken social contagion among nursing professionals. Therefore, findings from the study will have practical implications as to how healthcare institutions can lessen the occurrence of such behaviour (Lian et al. 2014) and determine the factors that increase its occurrence (Lugosi 2019). The conceptual model of the study is presented in Figure 1 below.

## 3 Study

### 3.1 Aims

The study's aim was to investigate the boundary conditions (i.e., personal norms and NFR) that strengthen or attenuate the social contagion effect among nursing professionals.

### 3.2 Design

The study used a cross‐sectional design to gather data among nursing professionals in Ghana (Bryman 2016). Although a cross‐sectional design limits a study from assessing the causal relationship among constructs (Bryman 2016), it offers new insight into a less investigated phenomenon, setting the pace for future investigation. Additionally, since this study dealt with nursing professionals within a large geographical area, the cross‐sectional design offered better logistical support than longitudinal and experimental designs. The study reporting followed the Strengthening Reporting of Observational Studies in Epidemiology (STROBE) (Von Elm et al. 2014).

### 3.3 Study Sample

The study targeted nurses in the Ashanti Region of Ghana because it has the largest representation of health facilities and healthcare workers (Ghana Investment Promotion Council 2023; Asante 2025). Currently, the region's healthcare facilities stand at 1654, spreading across the domains of primary, secondary and tertiary facilities (Ghana Health Service 2023). The study used a convenience sampling technique because the health facilities were sparsely spread within the region, resulting in difficulty accessing all the health facilities. Using convenience sampling is not new in nursing studies, as it has been deemed the most feasible, especially when a study has limited resources to access the entire population (Amarasena et al. 2015; Asante 2025). However, a purposive sampling approach was used to ensure that the selected health facilities better reflected the country's health care structure. The selected health facility was based on the categorisation of the Ghana Health Service and Ministry of Health: primary, secondary, and tertiary facilities. From the Ghana Health Service (GHS), facilities that fall under the respective categorisations (i.e., primary, secondary and tertiary) share the same characteristics regarding patient‐to‐doctor and patient‐to‐nurse ratios, equipment, and care delivery. The GHS categorises the country's health facilities into primary, secondary, and tertiary. Per the description of the GHS, a primary healthcare system becomes the lowest level and the entry point into the healthcare system (Ghana Investment Promotion Council 2023). They encompass health posts, clinics, and health centres, primarily providing basic healthcare services such as immunisation, malaria treatment, antenatal care, infant and young child feeding, etc. (Ghana Investment Promotion Council 2023). Secondary health facilities become the referral points for the primary facilities, especially for patients requiring specialists in cardiology, endocrinology, neurology, ear, nose and throat, and general surgery. They constitute the district and regional hospitals (Ghana Investment Promotion Council 2023). Tertiary healthcare comprises the higher levels of speciality in equipment and expertise. They become the referral points for secondary facilities and provide treatment and care for kidney dialysis, neurosurgery, cardiology, cardiac surgery interventions, and assisted reproductive technologies (Ghana Investment Promotion Council 2023). Accordingly, considering how the region's health facilities are dispersed along these categorisations, we purposely selected 10 clinics (i.e., primary health facilities), three polyclinics (i.e., primary health facilities), eight district hospitals (i.e., secondary health facilities), three other hospitals (i.e., secondary health facilities) and one tertiary health facility (i.e., the main tertiary hospital) to better reflect the country's health categorisation. Nurses working in these health institutions constituted the sampling frame for the sample selection.

However, power analysis was used to determine the sample size. According to Hintze (2008), the ideal power for a sample size estimation should be > 0.8 to minimise the risk of missing an actual effect in survey studies. Following this recommendation, the thresholds were set at a medium effect size of 0.15, a power of 0.95 and an alpha value of 0.05 (Kang 2015). Based on the chosen thresholds and with three predictor variables, the study required a sample size of 119 nurses. Though a sample size of 119 nurses was required to produce a medium‐sized effect, following methodological recommendations, it is always important to account for invalid responses in the sample size estimation (Hsu and Sandford 2007; Ward and Meade 2023).

Therefore, accounting for a projected 15% rate of invalid responses, a sample size of at least 137 was required. In this study, 293 valid questionnaires were collected and used for the analysis, considerably exceeding the minimum threshold. According to Ye et al. (2025), using an adequate sample size ensures acceptable statistical power, improves the validity of the model estimates, and strengthens the reliability of the results.

**FIGURE 2:**
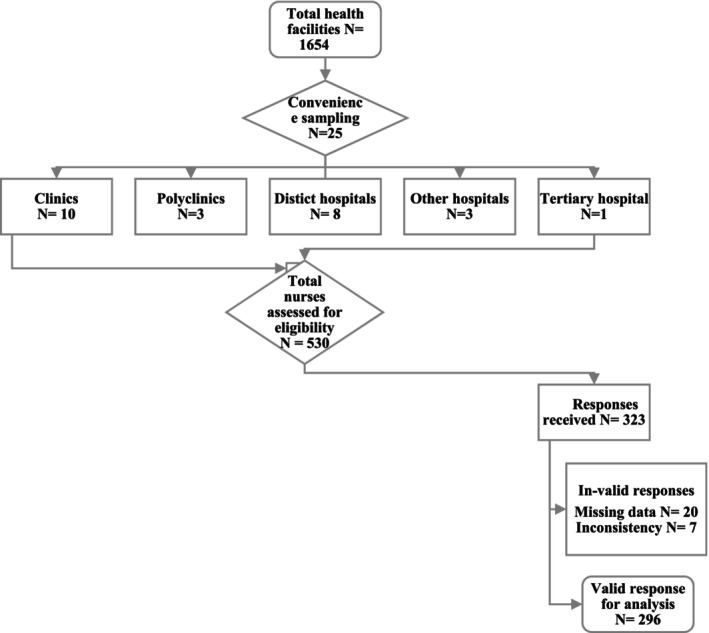
Sampling flow chart.

The criteria that guided a sample selection were a full‐time nurse with the appropriate license from the Nursing and Midwifery Council of Ghana. A sample was again required to have demonstrable working experience of at least one year post‐qualification. Nurses who met these criteria were included in the sampling frame in a health facility. Therefore, nurses who did not meet these criteria were excluded. The sampling chart flow for the sample selection is presented in Figure 2 below.

### 3.4 Data Collection

Before proceeding to the data collection, letters of introduction were sent to the respective health facilities' administrators and medical superintendents detailing the study's purpose. In‐person discussions were held with these bodies to explain the study's purpose and how the data would be collected and analysed. After obtaining institutional approvals, meetings were held with the nursing managers to allow them to seek further clarification about how the data collection would be organised and the levels it would take. Afterwards, a short debriefing was held with all the participants, explaining to them the purpose of the study and the benefits of the findings to the health sector. The nurses were guaranteed anonymity and again allowed to stop participating anytime they felt uneasy. The questionnaires were sent to the 530 nurses in person and via Google Forms. With Google Forms, a link to the questionnaire was sent to the nurses through a WhatsApp group, in which the head nurse served as the group manager. The head nurse was not granted access to either view the questionnaire or the responses, which assured the respondents that their responses were inaccessible to their colleagues or superiors. Again, a respondent was restricted to one entry to prevent multiple responses. In order to protect the anonymity of the nurses participating in the study, their emails were not collected.

After one week of no response, a reminder was sent to their WhatsApp group. Also, nurses who could not access the questionnaire link were given a printed copy to answer. After the agreed time, personal visits were made to the hospitals to collect the printed answered questionnaires. After receipt of the online and paper questionnaires, all responses were screened by the research team to identify issues of logical inconsistencies and missing data.

Out of the 530 distributed questionnaires, 323 responses were received, and after the screening done by the research team, 27 were excluded from the analysis due to significant missing data and logical inconsistency. In all, 296 responses were used for the analysis, giving a valid response rate of 58.8%. The data collection occurred between March 11 and May 12, 2024.

### 3.5 Measures

The scales used to measure the constructs were adapted from earlier scales. The superior's antisocial behaviour was measured with a five‐point Likert scale (1, Never to 5, Always). The study adopted the nursing manager's antisocial behaviour scale from Sackett (2002). With this scale, the items were not entirely modified; only the examples under each item were modified to reflect the healthcare setting. For instance, we maintained unsafe behaviour while its corresponding example was modified to reflect the setting better (e.g., failure to adhere to safety protocols; failure to learn safety procedures). The mean score for the superior antisocial behaviour was 2.2–3.5, indicating a never–always rating.

With the subordinate's antisocial behaviour, because the superior's antisocial behaviour was expected to diffuse to observers, it became the reverse of the superior's behaviour. The expected subjects required to perform this act now became the subordinates. An example item includes, ‘I frequently engage in unsafe behaviour (e.g., failure to adhere to safety protocols and failure to learn safety procedures)’. The mean score of this scale was from 2.3 to 4.3, indicating never to always rating. The Cronbach alpha value of the antisocial behaviour scale ranged from 0.755 to 0.761, confirming the constructs' high reliability (see Table 1).

**TABLE 1:** Reliability and validity checks of the constructs (*n* = 296).

Constructs	*α*	AVE	CR (rho_c)	Factor loading	Mean	SD
Superior antisocial behaviour	0.761	0.506	0.834			
Misuse of information (e.g., revealing confidential information or falsifying records)				0.599	3.562	0.661
Misuse of time and resources (e.g., wasting time, altering time cards, undertaking personal business during work)				0.609	3.591	0.631
Unsafe behaviour (e.g., failure to adhere to safety protocols; failure to learn safety procedures)				0.832	2.351	0.615
Poor attendance (e.g., unexcused absence or lateness; misapplication of sick leave) Poor quality work (e.g., intentionally slow or sloppy work)				0.787	4.291	0.626
Inappropriate verbal actions (e.g., verbally harassing patients and colleagues)				0.700	3.561	0.653
Inappropriate physical actions (e.g., physically attacking co‐workers; physical, sexual advances toward co‐workers)				0.796	3.311	0.605
Theft and related behaviour (e.g., theft of cash or property; giving away goods or services; misuse of employee discount)				0.821	2.223	0.721
Personal norm	0.867	0.600	0.900			
I feel a moral obligation to protect the universe				0.751	3.389	0.782
I feel that I should protect the values I stand for				0.776	4.621	0.673
I feel it is important that people, in general, protect the universe				0.788	4.212	0.583
Because of my values/principles, I feel obligated to behave socially responsibly				0.757	3.682	0.653
Need for recognition	0.871	0.723	0.912			
My promotion/success is reliant on what my manager says/does				0.773	4.581	0.794
My superiors will bail me out at all times				0.831	3.586	0.801
My leader would use his/her power to help me solve problems I find myself				0.892	4.182	0.793
My leader frequently recognises my contribution				0.899	4.321	0.555
Subordinate antisocial behaviour	0.755	0.508	0.836			
I frequently engage in inappropriate physical actions (e.g., physically attacking co‐workers and sexual advances toward co‐workers)				0.623	2.331	0.625
I have been found to have poor attendance (e.g., unexcused absence or lateness, misapplication of sick leave)				0.740	3.211	0.715
I frequently engage in unsafe behaviour (e.g., failure to adhere to safety protocols and failure to learn safety procedures)				0.812	3.561	0.634
I do not engage in quality work (e.g., intentionally slow or sloppy at work)				0.758	2.461	0.556
I often misappropriate time and resources (e.g., wasting time, altering time cards, and undertaking personal business during work)				0.608	2.356	0.452
Theft and related behaviour are easier to engage in at the workplace (e.g., theft of cash or property, giving away goods or services, misuse of employee discounts)				0.735	4.340	0.438
Engaging in inappropriate verbal actions is easier for me (e.g., verbally harassing patients and colleagues)				0.883	4.388	0.521

Abbreviations: *α*, Cronbach alpha; AVE, average variance extracted; CR, composite reliability; SD, standard deviation.

The personal norm scale came from the NAM (Asante et al. 2024). The items were not modified because of the NAM scales' broad applicability across industries (Asante et al. 2024). An example item includes ‘I feel a moral obligation to protect patient safety’. All the items were measured on a five‐point Likert scale (1, Strongly Disagree to 5, Strongly Agree). It recorded a mean score of 3.4–4.2, suggesting a neutral to agree rating (see Table 1). The study of Asante (2025) confirms the personal norm scale usability among nursing samples. Obtaining an alpha value of 0.867 on the PN scale, as reported in Table 1, affirms the items' high internal consistency.

The NFR scale was taken from Graen and Uhl‐Bien's (1995) leadership member exchange (LMX) scale. Without an established scale to measure NFR, we use the scale items from Graen and Uhl‐Bien's (1995) LMX scale, which aligned closely with the definition and the proposed rationale for subordinates' overdependence on leadership. For instance, Graen and Uhl‐Bien (1995) use these questions to measure leadership and subordinate exchanges and dependence: How would your leader use her power to help me solve problems? How would your leader ‘bail me out’ at her expense? And how do your leaders recognise your contribution? The participants were asked to indicate the extent to which they believed the imitation of superior antisocial behaviour resulted from the need to get approval or favour from the leader. An example of the scale is ‘My promotion/success relies on what my superior says/does’. All the items were measured on a five‐point Likert scale (1, strongly disagree to 5, strongly agree). NFR recorded a mean score of 3.5–4.5, suggesting an agreed rating. The NFR scale reported an alpha value of 0.871, indicating the scale's high reliability, as reported in the study of Graen and Uhl‐Bien (1995).

### 3.6 Statistical Analysis

First, the study's raw data were organised using Excel 2017. SPSS 28.0 and SmartPLS 4.1 were used for the statistical analyses. The categorical constructs were analysed with frequencies and percentages, whereas the continuous constructs were analysed with means ± standard deviations (mean ± SD). Further, to proceed with path analysis, the preliminary assessments of the scales were computed to confirm the scales' validity and reliability. With the preliminary assessment, convergent, discriminant and construct validity assessments were computed to assess the constructs' validity and reliability (Hair et al. 2019). According to Hair et al. (2019), to achieve convergent validity, the construct average variance extract (AVE) and cross‐loadings should be greater than 0.5 and 0.6, respectively. The results on the constructs' AVE and cross‐loadings are reported in Table 1. Composite validity assesses the internal consistency of the construct items, and a high construct validity is achieved when the composite reliability value is greater than 0.6. The heterotrait–monotrait (HTMT) ratio was utilised to assess discriminant validity. Emerging evidence has shown that the HTMT assessment criteria produce a more robust discriminant validity assessment than the often‐used measures, such as the Fornell–Lacker criterion and cross‐loadings (Henseler et al. 2015). To address multicollinearity issues, the outer model's variance inflation factor was assessed using the tolerance value threshold of > 0.1 and < 5 (Roberts and Thatcher 2009). Also, following the recommendations of Hair et al. (2019), the bootstrapping was computed for the confidence intervals of the HTMT. Results in the appendix confirm that none of the upper CI columns were above 1, suggesting that discriminant validity was not a serious issue (see Appendix A).

With the structural model assessment, the 10,000 bootstrapping algorithm was used to assess all the paths' level of significance. The parameters employed to assess the path coefficients are the *β*, SD, *t*‐values, *p*‐values, and confidence intervals. Guided by the recommendations of the extant literature, the *t*‐values ought to be greater than or equivalent to 1.96 at a 95% confidence level for the path to be considered significant (Hair et al. 2019). With the statistical significance of the path coefficients, their corresponding *p*‐value should be less than 0.05. Lastly, with the path coefficient confidence interval (i.e., lower bound–upper bound), the lower bound–upper bound should not be zero for a hypothesised relationship to be confirmed. The results of the path coefficients guided by these assessment indicators are presented in Table 4.

### 3.7 Common Method Bias

Considering the impact of common method bias (CMB) on the validity of the findings, procedural and statistical methods were employed. First, under the procedural measure, the measuring scales were randomised to minimise the possibility of respondents quickly guessing the expected relationship among the variables. Lastly, on the statistical method, using the IBM SPSS version 29, the Harman single‐factor test was computed (Podsakoff et al. 2003). After the estimation, the total difference explained by a single factor component stood at 38.7%, indicating that in this study, CMB has no severe impact (Malhotra et al. 2017).

### 3.8 Ethical Consideration

The study obtained consent from the health facilities' administrators and medical superintendents before collecting data. The study again received ethical approval from the Ethics Committee of the Appiah‐Menka University of Skills Training and Entrepreneurial Development, Ghana (Reference Number: 240115: 06/04/2024).

## 4 Results

### 4.1 Reliability and Validity Analysis

First, the convergent, discriminant and construct validity assessments were performed to assess the constructs' validity and reliability (Hair et al. 2019). With the convergent validity assessment, results in Table 1 reveal that the constructs' AVE and factor loadings met the minimum threshold of 0.6 for factor loadings and 0.5 for AVE (Hair et al. 2019). Additionally, on the construct factor loadings, apart from two items, each under superior and subordinate antisocial behaviour constructs, all the remaining items had factor loadings exceeding the recommended threshold of 0.7. Following the suggestions of the extant literature, these items were not deleted because their inclusion did not affect the scale's validity and reliability (Malhotra and Dash 2011). Results in Table 1 confirm that the scales achieve high reliability as their composite reliability (CR) scores were within the range of 0.834–0.913 (Hair et al. 2019). The HTMT was used as the assessment criterion for the scales' discriminant validity. According to Henseler et al. (2015), the HTMT criterion has been validated to produce a more robust discriminant validity assessment than the often‐used Fornell‐Larcker and cross‐loadings. Results in Table 2 confirm that all the scales achieved a stricter HTMT because all the constructs were under 0.85 (Henseler et al. 2015). Also, following the recommendations of Hair et al. (2019), the bootstrapping was computed for the confidence intervals of the HTMT. Results in the appendix confirm that none of the upper CI columns were above 1, suggesting that discriminant validity was not a serious issue (see Appendix A). The tolerance value and variance inflation factor (VIF) of each scale were within the suggested thresholds, tolerance value > 0.1 and VIF < 5, suggesting that multicollinearity is not a serious issue in this study (Roberts and Thatcher 2009).

**TABLE 2:** Heterotrait–Monotrait ratio (HTMT) (*n* = 296).

	NFR	PN	Sub B	Sup B	PN*NFR	PN*Sup B
NFR						
PN	0.755					
Sub B	0.809	0.525				
Sup B	0.761	0.622	0.718			
PN*NFR	0.655	0.733	0.306	0.417		
PN*Sup B	0.653	0.682	0.297	0.394	0.812	

Abbreviations: NFR, need for recognition; PN, personal norm; Sub B, subordinate antisocial behaviour; Sup B, superior antisocial behaviour.

Lastly, to proceed with the path analysis, the model's overall fitness was assessed using the standardised root mean square residual (SRMR) reported in SmartPLS (Henseler et al. 2014). SRMR assesses the variance between the observed and model‐implied correlation matrix (Pavlov et al. 2020). When it comes to the assessment of model fit in structural equation modelling, studies have shown that more accurate confidence intervals and tests of close fit are attained when using SRMR instead of RMSEA (Maydeu‐Olivares et al. 2018; Shi et al. 2020). The latter only provides accurate results in small models (Pavlov et al. 2020). From the estimation of Hu and Bentler (1999), an SRMR value of less than 0.10 or 0.08 is considered a good fit. Following the criterion of Hu and Bentler (Hu and Bentler 1999), the study model is deemed an acceptable fit because its SRMR value was < 0.080. The SRMR reported a value of 0.067 with a Chi‐square (*χ* ^2^) = 1160.572 and NFI of 0.958.

### 4.2 Sample Description

Results in Table 3 suggest that more of the surveyed nurses were between 20 and 40 years old, with 175 (59.1%) females. Again, most respondents had a diploma in nursing or a bachelor's degree as their educational level. Lastly, from the nurses sampled, 75 (25.3%) worked at the clinics, whereas 48 (16.2%) worked at the polyclinics (i.e., primary health facility). Also, 122 (41.2%) worked with the district and regional hospitals, with the remaining working at a tertiary health facility (i.e., 17.3%). Results from the study suggest that more nurses were engaged at the district and regional hospitals. The high representation of nurses at the secondary health facilities can be attributed to their roles in the country's healthcare delivery, being the main referral points for the primary healthcare facilities. Again, because they have better infrastructure and capability than the primary facilities, most nurses are likely to prefer working at a secondary facility to a primary facility.

**TABLE 3:** Respondents' demographic profile (*n* = 296).

Demographic variable	Category	Frequency	Per cent
Gender	Male	121	40.9%
	Female	175	59.1%
Age	20–30 years	78	26.4%
	31–40 years	160	54.1%
	41–50 years	58	19.5%
Response	Clinics	75	25.3%
	Polyclinics	48	16.2%
	District and regional hospitals	122	41.2%
	Tertiary hospital	51	17.3%
Level of education	Diploma in Nursing	144	48.6%
	Bachelor's degree	90	30.4%
	Master's degree	62	20.9%
Number of years they have worked as a healthcare worker	1–5 years	121	40.9%
	6–10 years	98	33.1%
	More than 10 years	77	26.0%

### 4.3 Structural Model

The study model *R*‐squared values were within the range of 0.632 to 0.634, representing moderate predictive power. The model's effect size (*f* ^2^) is between 0.294 and 0.376, indicating a medium‐sized effect (Cohen 1988). Lastly, with the model's predictive relevance, the *Q* ^2^ values exceeded the recommended threshold of > 0, confirming the model's predictability (Hair et al. 2016).

### 4.4 Relationship Testing

The model was examined further to understand the relationship between the constructs. H1 measures the relationship between Sup.B and Sub.B. The results in Figure 2 confirm H1 as it reports a positive standardised path coefficient of 0.698 (*p* < 0.001), suggesting that superior antisocial behaviour positively influences junior nurses' antisocial behaviour. Likewise, H2 gains support from the sample as the results in Figure 2 report a significant positive relationship between superior antisocial behaviour and NFR (Sup.B → NFR, 0.451, *p* < 0.001). Hypothesis H4 measures how the NFR influences nurses' subordinate antisocial behaviour. H4 was affirmed as the results in Table 4 suggest a significant positive relationship between the NFR and subordinate antisocial behaviour (NFR → Sub.B, 0.544, *p* < 0.001). The hypothesised relationship between PN and Sub.B was not supported, indicating that personal norm did not influence subordinates' antisocial behaviour (PN → Sub.B, 0.123, *p* > 0.05).

**TABLE 4:** Standardised direct and indirect effects for the hypothesised relationship (*n* = 296).

	Coeff (*β*)	Sample mean	SD	*T*‐statistics	*p*	95% CI	Conclusion
Direct effects							
H1: NFR → Sub B	0.544	0.538	0.071	7.653	0.001	[0.400, 0.678]	H3 supported
PN → Sub B	0.123	0.127	0.111	1.105	0.269	[0.100, 0.340]	Not supported
Sup B → Sub B	0.698	0.702	0.061	11.394	0.001	[0.582, 0.828]	H1 supported
Sup B → NFR	0.451	0.456	0.059	7.699	0.000	[0.336, 0.562]	H2 supported
PN → NFR	0.211	0.211	0.087	2.422	0.015	[0.034, 0.380]	Supported
Indirect effects							
PN*SupB → Sub B	−0.096	−0.092	0.024	4.075	0.001	[−0.141, −0.052]	H6 supported
PN*Sup B → NFR	−0.177	−0.171	0.040	4.435	0.001	[−0.252, −0.099]	H5 supported
Sup B → NFR → Sub B	0.245	0.245	0.025	5.426	0.001	[0.018, 0.220]	H3 supported
**Structural model**	***R*** ^**2**^	**Adj. *R*** ^**2**^	***F*** ^**2**^	***Q*** ^**2**^	**RMSE**		**MAE**
NFR	0.634	0.627	0.376	0.638	0.638		0.486
Sub B	0.632	0.623	0.294	0.493	0.717		0.568

Abbreviations: Adj. *R* ^2^, Adjusted *R*‐square; *F* ^2^, *f*‐square; MAE, mean absolute error, *p*‐values < 0.001; NFR, need for recognition; PN, personal norm; *Q* ^2^, Q‐square (i.e., predictive relevance); *R* ^2^, *R*‐square; RMSE, root mean square deviation; SD, standard deviation; Sub B, subordinate antisocial behaviour; Sup B, superior antisocial behaviour.

### 4.5 Indirect Effect

**FIGURE 3:**
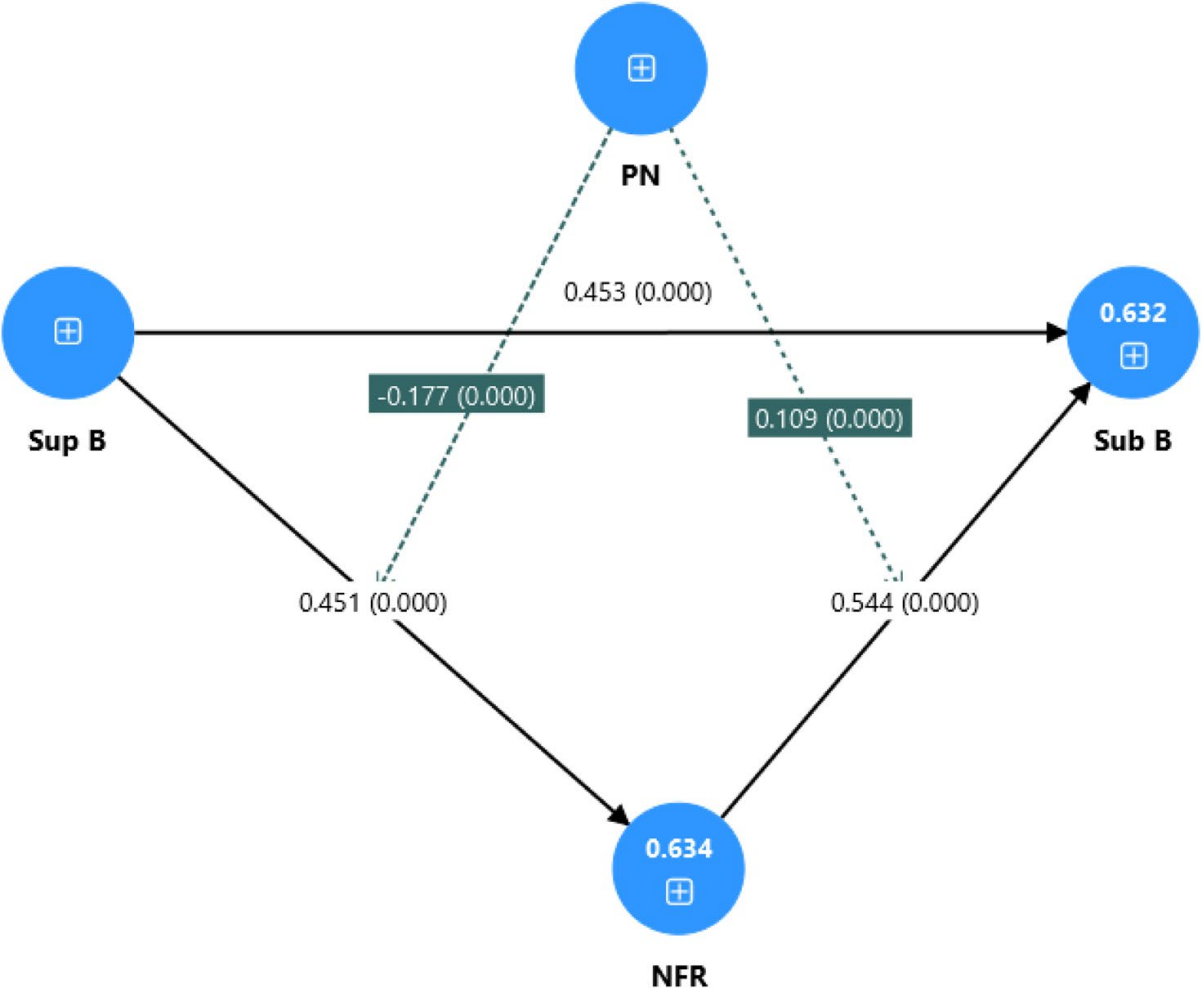
Structural model for relationship testing. NFR, need for recognition; PN, personal norm; Sub B, subordinate antisocial behaviour; Sup B, superior antisocial behaviour. Negative relationship. Values in brackets *p < 0.05. **p < 0.01. ***p < 0.001.

**FIGURE A1:**
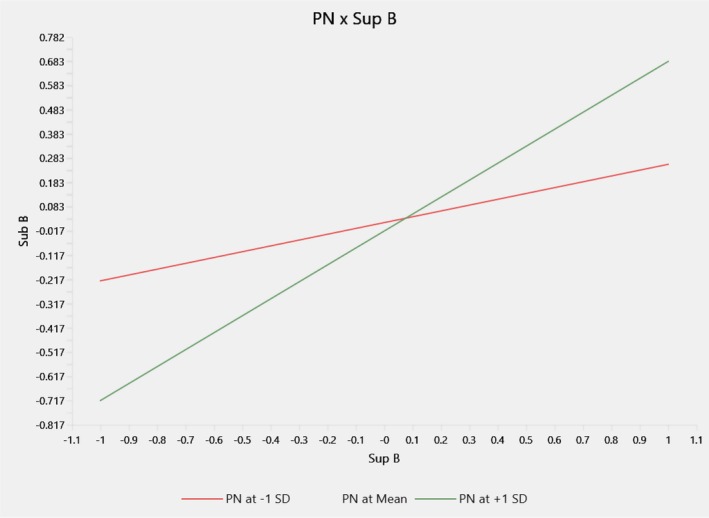
Moderated interaction effect of personal norm and subordinate antisocial behaviour on subordinate behaviour. PN, personal norm; Sub B, subordinate antisocial behaviour; Sup B, superior antisocial behaviour.

**FIGURE A2:**
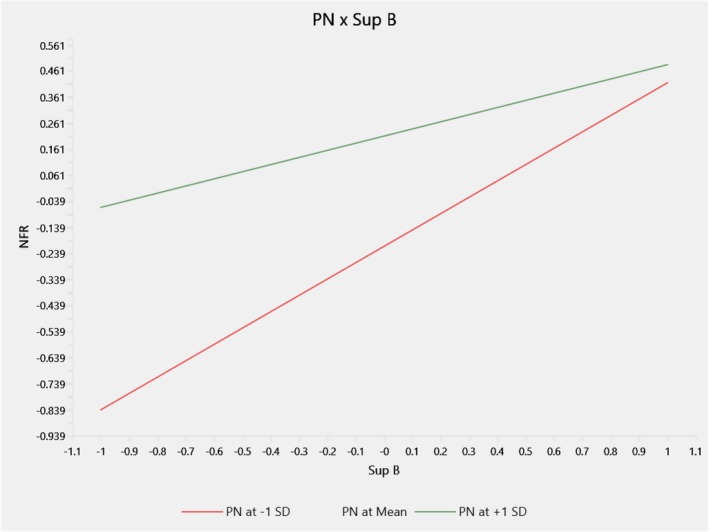
Moderated interaction effect of personal norm and subordinate antisocial behaviour on need for recognition. NFR, need for recognition; PN, personal norm; Sup B, superior antisocial behaviour.

The study was guided by the recommendations of Cepeda et al. (2017) to test the hypothesised indirect effects in the model. Results in Figure 3 confirmed that the NFR mediated the relationship between superior and subordinate antisocial behaviour (Sup.B → NFR → sub.B, *p* < 0.001). H3, therefore, gains support from the sample. Again, with the moderation analysis, results in Table 4 confirm that personal norm negatively moderated the relationship between superior antisocial behaviour and NFR (PN*Sup.B → NFR, −0.177, *p* < 0.001), confirming H5. This suggests that as the nurse's personal norm increases, the contagion effect expected between superior antisocial behaviour and NFR weakens. Similarly, PN inversely moderated the relationship between Sup.B and Sub.B (PN*Sup.B → Sub.B, −0.096, *p* < 0.001), indicating that a solid personal norm reduces the impact of superior antisocial behaviour on subordinate antisocial behaviour (see Figure 3). H6 gains support from the sample. Additionally, we recognise that the interaction coefficient may not be adequate to confirm the investigated moderation hypotheses, as the direction of the interacted effects is better observed in a graphical presentation (Hoetker 2007). Accordingly, to support our interpretation, the interaction was plotted at ±1 standard deviation employing mean‐centred values (Cohen et al. 2003). The results of the interaction effect (i.e., PN*Sup.B → Sub.B) and (i.e., PN*Sup.B → NFR) are plotted in Figures A1 and A2, respectively (see Appendix B).

## 5 Discussion

The study's primary purpose was to investigate the underlying conditions that strengthen or attenuate the social contagion effect among nursing professionals. This research tested several relationships to expand the conversation about whether the proposed relationship between social contagion and employee outcomes is permanently unidimensional (Mayer et al. 2010; Cialdini et al. 2021). Results from the relationship analysis reveal several outcomes suggesting that leadership antisocial behaviour's influence on subordinates may not always be uniform. First, with the direct effect analysis, H1, H2 and H4 gain support from the study sample. H1 suggested that the antisocial behaviour of senior nurses will spill over to junior nurses. The results from H1 strengthen the social information processing theory (SIP) (Salancik and Pfeffer 1978), as it suggests that individuals inform their sense of reality from the signals gathered from their social environment.

Likewise, in the health setting, nursing managers play several responsibilities, spanning managerial and clinical roles within specific departments. Junior nurses often perceive them as management representatives because of their intermediary role between nursing teams and hospital management. Accordingly, actions performed by nursing managers are likely to be perceived as acceptable, and they create the expectation that their imitation will lead to tremendous success (Salancik and Pfeffer 1978). The result from the study is consistent with the findings of Strang et al. (2016), as their work observed that undesirable feelings received within a social setting activated unkind treatment among subordinates. Although the sample of Hoobler and Hu (2013) is not comparable to this study, their results affirm this study's findings. Findings from the study of Hoobler and Hu (2013) observed that mistreated individuals tend to release their bad experiences on unconnected others, such as juniors, colleagues, or even close family members, activating a contagious spread of hostile behaviour in interpersonal exchange.

Furthermore, results from H2 imply that nursing managers' antisocial behaviour strengthens junior nurses' NFR. Generally, because nursing managers also perform administrative duties such as scheduling, resource management, and institutionalisation of career advancement for junior nurses, they are well admired within their departments (Asante 2025). Therefore, the significant relationship between nursing managers' antisocial behaviour and the NFR reinforces the interdependencies between leadership–subordinate exchanges, where subordinates depend on their leaders for decision outcomes. Likewise, H3 confirms a significant positive relationship between the NFR and junior nurses' antisocial behaviour. What can be inferred from this result is that, because nursing managers possess power and credibility in a healthcare setup, junior nurses' dependence on their managers for advancement and favour tends to be heightened (Offergelt et al. 2019). The desire to gain favourable responses from superiors will, in effect, strengthen superiors' antisocial behaviour on subordinates' NFR.

Additionally, by investigating the indirect effects (i.e., moderating and mediating), the study departs from the extant literature by identifying the boundary conditions that intensify or weaken social contagion among nursing professionals. The results on H3 reveal that the NFR mediates the relationship between nursing managers' antisocial behaviour and subordinate antisocial behaviour. The results suggest that junior nurses' desire to achieve favourable responses from their superiors strengthens their decision to copy their superiors' antisocial behaviour. Findings from the study underpin the theoretical lens of the social identity theory (SIT) among nursing professionals, suggesting that an individual who excessively identifies with their leaders mainly because of their dependence on the leader/unit may feel compelled to pursue the leader's ideals (Umphress and Bingham 2011; Lee et al. 2015). Therefore, the social contagion effect of antisocial behaviour tends to be more consistent when subordinate career advancement and satisfaction heavily depend on the leader's pronouncements. Results from the study are consistent with the arguments of Lindenberg et al. (2021) and Ogulmus et al. (2024), as they contended that the effect of social contagion on individual behaviour becomes very substantial, particularly when the observer perceives the architect as powerful or the leading decision maker.

With the moderating analysis, results from H5 and H6 suggest that high personal norms weakened the relationship between superior antisocial behaviour, subordinate behaviour and the NFR. The results point out that the extant literature over‐emphasises that social contagion and employee outcomes will always lead to a homogenous outcome, but may not always be so (Mayer et al. 2010; Cialdini et al. 2021). The results corroborate the views of Krupka and Weber (2013) that, so far, a nurse may care more about the social suitability of their actions (Krupka and Weber 2013); their norms and values can thwart the spillover of leadership influence on junior nurses' antisocial behaviour (Kish‐Gephart et al. 2010). The results suggest that because personal norms and values enable a person to define what is good, worthy and critical in their life (Kmieciak 2024), they become the far‐reaching and trans‐situational goals that act as a signpost in a person's endeavours (Sagiv et al. 2017). Therefore, the inverse moderation role of personal norms in the superior antisocial behaviour and subordinate behaviour relationship suggests that nurses who regard their duty to nursing and patient care as a moral call will not be easily persuaded to copy the bad habits of their nursing managers.

Also, results from the study revealed that high personal norms reduced the impact of superior antisocial behaviour on the NFR. The results indicate that the influence of superior antisocial behaviour on subordinates' NFR is reduced when they have a high personal norm (i.e., they have a strong feeling of upholding their values). The more assertive nurses perceive their duties as a moral obligation, the less they are moved to emulate the same destructive behaviours of their superiors to be in their good books. The results from the study expand the non‐homogeneity of the social contagion effect in the extant literature by highlighting that a person can be differentially impacted by organisational ideas and practices based on the non‐alignment between their personal and organisational actions and values (Cable and Edwards 2004). Accordingly, the social contagion effect of superior antisocial behaviour may not always be, even as suggested earlier in the extant literature (Mayer et al. 2010; Cialdini et al. 2021; Liu et al. 2021).

### 5.1 Strengths and Limitations

The study offers several strengths. First, the study expands the existing literature on social contagion, particularly among nursing professionals, as only a few studies have been done among the nursing sample. Again, in response to the nonuniformity of leadership's influence on subordinate behaviour, the study investigated the underlying factors that could intensify or diminish social contagion among nursing professionals. Drawing on the theoretical lens of the SIP and NAM, we included two social constructs (i.e., the NFR and personal norm) to understand whether the social contagion among nursing professionals will remain the same. Results from the study suggest that the social contagion effect on the observer increases, particularly when the subordinate reckons that their career progression decision relies on their imitation of the leaders' antisocial behaviour.

In contrast, the impact of social contagion decreases, especially when the observer has a strong personal norm (i.e., regards their nursing duties as a moral obligation). The study results imply that the social contagion effect of superior antisocial behaviour on subordinates may not always be as strong as suggested in the literature (Mayer et al. 2010; Cialdini et al. 2021) but may be determined by individual‐level factors such as norms, values and subordinate high dependence on the superior for survival. Similar to other studies, the study is not void of limitations. The study findings are based on the nurses' shared perceptions about their superiors' antisocial behaviour. As self and other‐reported leadership practices could fluctuate, it would be worthwhile to investigate whether and to what extent superior versus subordinate‐rated antisocial behaviour would affect the study conclusions. Specifically, the agreement or inconsistency between these two‐way assessments (i.e., 360° leadership and subordinate ratings) could expand the extant literature on whether the impact of social contagion lies in the eye of the observer or is grounded in actual behaviour.

Additionally, because of the limitation of cross‐sectional data in making robust causal inferences among two or more variables, a longitudinal study among these variables in future studies is key. Finally, another limitation is that the study did not explore the impact a sample health facility's status (i.e., primary, secondary and tertiary institution) had on their assessment or perception of their superior's antisocial behaviour. Future studies should investigate how these healthcare facility characteristics impact observer perception of superior antisocial behaviour and solidify the spillover effect of the antisocial behaviour.

## 6 Conclusion

The results provide insight to nursing management on the essence of providing positive experiences to junior nurses as they reuse these experiences as a signpost for their practices in future exchanges. The study findings reveal that nursing managers' antisocial behaviour significantly influenced nurses' antisocial behaviour, suggesting that nursing managers should serve as good role models, as their actions and behaviour shape their subordinates' behaviours.

Also, results from the study indicate that the impact of superior antisocial behaviour on junior nurses may not translate into the same degree of effect, especially when the nurse perceives her role as a call to duty. The results suggest that by enhancing the nursing curricula and training with topics on values and principles, especially during in‐school and on‐the‐job training, nurses can recognise the importance of adhering to professional principles and standards even when their superior fails to provide the needed leadership experience.

## Consent

The authors have nothing to report.

## Conflicts of Interest

The authors declare no conflicts of interest.

## Data Availability

The data are not publicly available due to privacy or ethical restrictions. The data that support the findings of this study are available on request from the corresponding author.

## Appendix A

Bootstrapped Discriminant Validity Heterotrait–Monotrait Ratio (HTMT) Method‐HTMT Confidence Interval Bias Corrected.

**Table:**

	Original Est.	Bootstrap mean	Lower	Upper
NFR → PN	0.755	0.752	0.531	0.829
NFR → Sub B	0.809	0.806	0.486	0.601
NFR → Sup B	0.761	0.761	0.641	0.810
NFR → PN*NFR	0.655	0.655	0.587	0.807
PN → Sub B	0.525	0.525	0.427	0.925
PN → PN*NFR	0.733	0.733	0.815	0.941
Sub B → Sup B	0.718	0.709	0.563	0.881
Sub B → PN*NFR	0.306	0.306	0.357	0.557
Sup B → PN*NFR	0.417	0.415	0.660	0.872

Abbreviations: NFR, need for recognition; PN, personal norm; Sub B, subordinate antisocial behaviour; Sup B, superior antisocial behaviour.
